# A Randomized Controlled Trial Comparing the Tolerability and Efficacy of Maribavir vs. Valganciclovir for Cytomegalovirus (CMV) Prophylaxis in High-Risk Kidney Transplant Recipients: Study Protocol

**DOI:** 10.7759/cureus.91110

**Published:** 2025-08-27

**Authors:** Hannah Culpepper, Morgan Overstreet, Karim Soliman, Michael Casey, Teresa Rice, Kaylie Lively, Joseph Scalea, John McGillicuddy, Neha Patel, David Taber

**Affiliations:** 1 Clinical Research, Medical University of South Carolina, Charleston, USA; 2 Transplant Nephrology, Medical University of South Carolina, Charleston, USA; 3 Nephrology, Medical University of South Carolina, Charleston, USA; 4 Transplant, Medical University of South Carolina, Charleston, USA; 5 Pharmacy, Medical University of South Carolina, Charleston, USA

**Keywords:** cytomegalovirus (cmv), drug randomization, immunosuppresion, kidney transplantation recipients, randomized clinical trial

## Abstract

Background

Cytomegalovirus (CMV) infection remains a significant problem in kidney transplantation despite advances in screening, monitoring, therapeutics, and management. Although universal prophylaxis with antiviral therapy has significantly reduced the risk of early CMV infection and disease, late-onset CMV is still common and can be difficult to clinically manage in high-risk patients. A recent systematic review showed that with antiviral prophylaxis, early CMV infection occurred in only 6% of kidney recipients, and late infection occurred in more than one in six patients.

The two antiviral prophylaxis medications this study is comparing, valganciclovir (VGC) and maribavir, are highly effective at preventing CMV infection. In studies using valganciclovir, the reported occurrence of leukopenia is 20%-40%, and neutropenia is 10%-30%. In studies using maribavir, the reported occurrence of neutropenia was 4%-5% versus 15%-18% in valganciclovir patients. With appropriate dosing, maribavir appears to have similar efficacy to valganciclovir in treating current and preventing future CMV infection with a significantly reduced rate of neutropenia.

Methods

Maribavir IIR is a 12-month, single-center, open-label, randomized controlled trial enrolling 70 patients (35 in each arm) examining the difference in preventing CMV infection while specifically assessing the tolerability of the two antiviral prophylactic medications. The trial is currently in the follow-up phase, with the first patient enrolled in November 2023 and enrollment concluding in June 2024.

Discussion

The primary objective of this study is to assess the tolerability of maribavir versus valganciclovir (VGC) prophylaxis in adult kidney transplant recipients at high risk of CMV infection (D+/R- or thymo use if R+). This was done by assessing the incidence of leukopenia in the two arms, the occurrence of CMV infection despite prophylaxis, the impact of these medications on healthcare utilization and costs, and any outcome differences associated with race and sex. In this preliminary report, we describe the study design, methods, aims, and outcome measures that will be utilized in the ongoing Maribavir IIR clinical trial.

Trial registration

The trial is registered at ClinicalTrials.gov NCT06034925: https://www.clinicaltrials.gov/study/NCT06034925.

## Introduction

Despite substantial advances in screening, monitoring, therapeutics, and management, cytomegalovirus (CMV) infection remains a significant problem in kidney transplantation. CMV is considered by clinicians to be one of the most important post-transplant infections [1-4]. Although prophylaxis with potent antiviral therapy has significantly reduced the risk of early CMV infection and disease (≤ 6 months), late-onset CMV (> 6 months post-transplant and once prophylaxis is complete) is still common and can be difficult to clinically manage. This is particularly true in high-risk patients-those receiving rabbit antithymocyte globulin (rATG) induction and those that are sero-mismatched (donor positive/recipient negative; D+/R-) [1]. A recent systematic review demonstrated that with antiviral prophylaxis, early CMV infection occurred in only 6% (95% CI 1 to 31%) of kidney transplant recipients, while late infection occurred in more than one in six patients (17%, 95% CI 2 to 29%) [4]. The results of the IMPACT study, a randomized controlled trial comparing 100 days to 200 days of oral VGC therapy in D+/R- patients, highlight the continued issue of late CMV infection. Although patients randomized to the 200 days of therapy had a significant reduction in CMV disease, the 12-month incidence of CMV disease was still 16.1% in these patients [5]. 

VGC antiviral prophylaxis is highly effective at preventing CMV infection, but it has numerous limitations. Of particular concern are the high rates of cytopenias, most notably, leukopenia and neutropenia. This often leads to a clinical conundrum, whereby it is unclear if the low white blood cell (WBC) count is due to VGC therapy itself, a breakthrough CMV infection, or other drug therapy. Oftentimes, this clinical dilemma causes additional laboratory testing, reductions in VGC and/or mycophenolate therapies, and, in severe cases, the administration of filgrastim (Neupogen®, G-CSF) [6-10]. In studies using VGC for CMV prevention, the reported incidence of post-kidney transplant leukopenia is 20%-40%, while neutropenia is observed in over 10%, reaching up to 30% [6-12].

Maribavir has been evaluated for the prevention of CMV infection in phase II and phase III trials among allogeneic stem cell transplant recipients. At six months post-transplant, the rates of CMV infection were not different between the treatment and placebo arms, although the rates of pp65 antigenemia were lower in the maribavir group. However, these studies used a low dose of 100 mg PO BID. In subsequent studies within solid organ transplantation, maribavir dosed at 400-1200 mg PO BID was equally efficacious to VGC for the treatment of CMV infection. Of note and importance to this proposal, the rates of neutropenia were 4%-5% in the maribavir-treated patients vs. 15%-18% in valganciclovir-treated patients. Thus, at an appropriate dose, maribavir appears to have similar efficacy to VGC in preventing and treating CMV infection with a significantly reduced incidence of neutropenia. Currently, there is a lack of randomized controlled trials assessing the safety and efficacy of adequately dosed (≥ 400 mg PO BID) maribavir for the prevention and treatment of CMV infection in solid organ transplant recipients [13-21].

This article was previously posted to the Research Square preprint server on July 19, 2024. 

## Materials and methods

Objectives

The primary aim is to compare and assess the tolerability and efficacy of the two antiviral regimens, as determined by the incidence of leukopenia in those randomized to maribavir vs. VGC prophylaxis in adult kidney transplant recipients at high risk of CMV infection. Additionally, the study will evaluate the impact of maribavir versus VGC prophylaxis on healthcare utilization and costs in adult kidney transplant recipients at high risk of CMV infection.

The exploratory aims for the study are to assess the impact of maribavir vs. VGC prophylaxis in adult kidney transplant recipients at high risk of CMV infection on patient-reported outcomes for quality of life and satisfaction, and assess any differences in leukopenia, efficacy, healthcare utilization, and patient-reported outcomes by race and sex in patients randomized to maribavir vs. VGC prophylaxis in adult kidney transplant recipients at high risk of CMV infection.

Trial design

**Figure 1:**
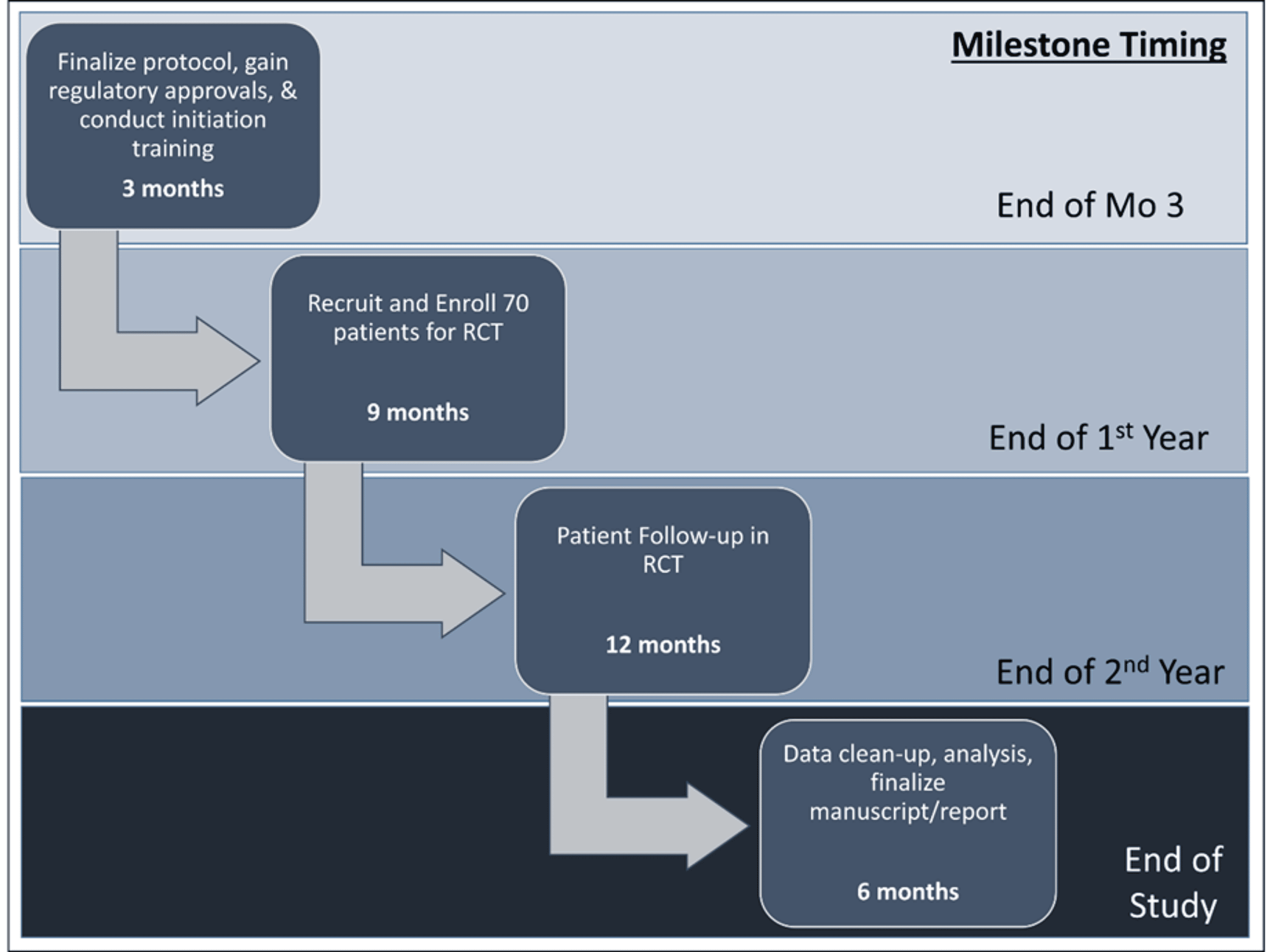
Milestone timing The image is created by the author.

This will be a 12-month, single-center, open-label, randomized controlled clinical trial enrolling 70 total patients (35 in each arm). Given the limited sample size and single-center design, this is a small, exploratory trial with limited power to detect significant differences between treatment arms for rates of CMV infection and other clinical endpoints (acute rejection, graft loss, death). The study is powered to detect clinically meaningful and statistically significant differences in the tolerability of the two regimens, as defined by the incidence of leukopenia accompanied by a reduction in total mycophenolate dose or VGC dose (see power/sample size section). Patients will be randomly assigned to receive VGC 900 mg PO daily (this is considered standard of care (SoC), adjusted for renal function) for 3-6 months post-transplant (based on CMV donor/recipient (D/R) serostatus as detailed below) or maribavir 400 mg PO BID for 3-6 months post-transplant (based on CMV D/R serostatus as detailed below) in addition to acyclovir 400 mg PO BID for one month post-transplant (investigation arm; the addition of acyclovir to maribavir is considered standard of care (SoC) for herpes simplex virus (HSV) prophylaxis). Patients who are D+/R- will receive six months of prophylaxis, while all other CMV serostatuses will receive three months of prophylactic therapy (per our institutional CMV protocol). All other medications, including immunosuppression and additional anti-infective prophylaxis, and follow-up assessments and clinic visits will be per SoC. The only additional procedure specific to this study is that patients will be self-administered two qualities of life surveys at 3-, 6-, and 12-month post-transplants, which can be completed online during a routine clinic visit or at home. Figure 1 displays the anticipated study duration and milestone timing, beginning with approval and study training and concluding with finalization of data and reporting.

Sample size

This study is adequately powered to detect a clinically meaningful and statistically significant difference in the incidence of clinically significant leukopenia in those randomized to maribavir vs. VGC under most incident rate scenarios. For this study, clinically significant leukopenia is defined as a WBC count of less than 3,000 mL AND a reduction in the dose of mycophenolate or VGC. Based on a retrospective analysis of internal data, the expected incidence of clinically significant leukopenia in the VGC arm is between 33 and 35%. To support this incident rate, we conducted a retrospective analysis of our internal data to fully assess the incidence of clinically significant leukopenia. In this analysis, we included 446 adult kidney transplant recipients transplanted at MUSC between 2007 and 2015. We chose this time period, as we had comprehensive data on induction, WBC counts, and mycophenolate dosing to allow for assessment of the incidence of clinically significant leukopenia. Of these patients, 127 (28%) received rATG induction. The incidence of WBC < 3000 with and without mycophenolate dose reduction is detailed in Table 1, which was assessed within one year of transplant. This data matches our expected incidence of leukopenia in the VGC arm of 35%. This is because patients in the proposed study will receive rATG or be CMV D+/R- and receive six months of VCG/maribavir, and be at higher risk of leukopenia.

**Table 1:** Incidence of WBC <3000 within one year of transplant WBC: White blood cell, MMF: Mycophenolate mofetil

Induction Agent	N	WBC <3K	WBC <3K and MMF Dose Reduction
Basiliximab	319	36.7%	21.3%
rATG	127	56.7%	34.6%

The incidence of clinically significant leukopenia (WBC < 3000 AND dose reduction in mycophenolate) is difficult to assess in the maribavir arm, as this specific issue is not reported in the published data. However, there are several studies that can be used to estimate this incidence for power/sample size purposes. In a study conducted in liver transplant patients known to be at higher risk for cytopenias than kidney recipients, the incidence of neutropenia was 6% in the maribavir arm [16]. In a phase III prophylaxis study in allogeneic stem-cell transplants, who are at very high risk for cytopenias, the incidence of neutropenia was similar between the maribavir and placebo arms, and the average WBC count was 4200 in the placebo arm and 4400 in the maribavir arm [15]. Thus, maribavir does not appear to induce or worsen leukopenia, and a 5% incidence is a reasonable expectation in the maribavir treatment arm for estimating sample size and power.

To meet at least 80% power, given an incidence rate of 33%-35% in the VGC arm and 5%-6% in the maribavir arm, 32 patients per arm are required, assuming two independent samples, a two-sided a=0.05, and using the chi-square test. This sample size will be increased to 35 patients per arm to address an expected dropout/lost-to-follow-up rate of 3%-5% in each arm. Thus, 70 total patients, 35 in each arm, will provide adequate power to assess the primary aim of this study. SAS 9.4 (SAS Institute, Cary, NC) was utilized to conduct sample-size estimations (Proc Power). 

For comprehensiveness, we conducted 17 different power/sample size analyses to assess different incident rates of clinically significant leukopenia in both the VGC arm and maribavir arm. These ranged from 30%-35% in the VGC arm and 5%-10% in the maribavir arm. With a sample size of 70 patients, 35 in each arm, we have adequate power to determine statistically significant differences between arms in 71% of these incidence rate scenarios. Table 2 displays the varying power estimates from 0.5-0.95 based on the 12 most likely incident rate scenarios for leukopenia with VGC and maribavir and the estimated required sample sizes needed for these power calculations. Based on these incidence rate estimates, adequate power is achieved for sample sizes between 50 and 75 patients in these scenarios. Required sample sizes beyond 70 patients are not common and appear to depend more on higher rates of leukopenia in the maribavir arm (increasing above 7%) vs. lower rates of leukopenia in the VGC arm (decreasing below 32%).

**Table 2:** Leukopenia incidence rate estimates between valganciclovir and maribavir

Incidence of Leukopenia in the Valganciclovir Group	Incidence of Leukopenia in the Maribavir Group	N Total	Actual Power	N Total with 5% Inflation Due to Dropouts	N Total with 3% Inflation Due to Dropouts
0.35	0.05	52	0.81	55	54
0.35	0.06	58	0.813	61	60
0.35	0.07	62	0.801	66	64
0.35	0.08	70	0.809	74	73
0.35	0.09	76	0.802	80	79
0.35	0.10	84	0.802	89	87
0.34	0.05	54	0.805	57	56
0.33	0.05	58	0.812	61	60
0.32	0.05	60	0.805	63	62
0.31	0.05	61	0.808	65	63
0.30	0.05	68	0.809	72	71
0.34	0.06	60	0.806	63	62
0.33	0.07	70	0.803	74	73
0.32	0.08	84	0.807	89	87
0.31	0.09	100	0.803	105	103
0.30	0.10	122	0.803	129	126
0.33	0.06	64	0.809	68	66

Participants and recruitment

Patients who are screened and meet criteria will be approached by study personnel for informed consent within seven days of kidney transplantation. This will occur during the index hospitalization for the transplant. Participants eligible for inclusion in the study must be at least 18 years old at the time of their transplant and within seven days post-transplant. They must have received at least one dose of rATG induction therapy or be D+/R- CMV serostatus. Individuals with impaired decision-making capacity are also eligible to participate.

Exclusions from the study criteria include individuals under 18 years old at the time of transplant, recipients of pancreas, liver, heart, or lung transplants, and those currently using any investigational or non-Food and Drug Administration-approved medications. Additionally, patients who are D-/R-or at low risk of contracting CMV will not be included.

Withdrawal or dropout criteria

Patients who are lost to follow-up, withdraw, or are taken off the study drug will not be replaced. Participation in this study is completely voluntary. Patients may withdraw at any time during the study. Treating physicians or providers may also decide to withdraw patients from the study if there are any safety concerns. Patients who choose to withdraw will be asked if they are willing to allow the data already collected on them to be included in analyses. Inclusion in the data analysis is also completely voluntary. If patients agree, their data will be censored at the time of withdrawal, and these patients will be included in the intent-to-treat analyses.

Assignment of interventions

After discussing the details of the study, providing ample time, and gaining informed consent, patients will be randomized in a 1:1 fashion (blocks of 10) to either the treatment or control arm.

Implementation

Once the patient has received at least one dose of CMV prophylaxis medication, they will be included in the study in an intent-to-treat manner. 

Intervention

Patients will be randomized to receive either maribavir 400 mg PO BID or VGC 900 mg daily (standard of care dose, adjusted for renal function) for CMV prophylaxis for 3-6 months post-transplant. Those who are CMV serostatus D+/R- will receive six months of antiviral therapy; all others will receive three months of therapy. The current gold-standard therapy is VGC for 3-6 months of therapy. Those randomized to maribavir will also receive one month of acyclovir 400 mg PO BID for HSV prophylaxis (standard of care dose). Beyond these interventions, all follow-up care and laboratory monitoring will be conducted as per standard of care (SoC) protocols. All immunosuppression will be given and dosed as per SoC protocols. There will be no additional visits or laboratory measurements as part of this study.

Follow-up

Participants will be monitored monthly for the first six months through chart review, and then every three months after that until they have reached 12 months, in which case they will be considered to have completed the study. Starting at baseline, participants will be contacted at 3, 6, and 12 months to complete the PROMIS SF QOL (Patient-Reported Outcomes Measurement Information System Short Form Quality of Life) and Patient Experience with Treatment and Self-Management questionnaires. They will complete the questionnaires a total of 4 times. Table 3 shows the schedule of assessments once the participant has signed consent and is deemed enrolled in the trial.

**Table 3:** Schedule of assessments CBC: Complete Blood Count, BMP: Basic Metabolic Panel, CMV: Cytomegalovirus, HRQOL PROMIS: Health-Related Quality of Life Patient-Reported Outcomes Measurement Information System 1. Within 48 hours of transplant. 2. Includes testing for CMV, Epstein-Barr, hepatitis B & C, and HIV for donor and recipients. 3. Includes assessments of adverse events, antiviral drug doses, allograft rejection, infection, need for dialysis, hospitalization, ED visits, graft loss, and all immunosuppressant medications and doses [15,16].

Assessments		Pre-transplant	Day post-transplant							Week post-Txp		Month post-transplant		
Time Frame			1	2	3	4	5	6	7	2	4	3	6	12
RANDOMIZATION			X											
History and Physical^1^		X							X		X	X	X	X
Vital Signs		X	X			X			X	X	X	X	X	X
Serologies^2^		X												
Laboratory Assessment	CBC and BMP	X	X			X			X	X	X	X	X	X
	Tacrolimus levels				X				X	X	X	X	X	X
CMV Infection Assessment									X		X	X	X	X
Clinical assessment^3^									X	X	X	X	X	X
HRQOL PROMIS Survey												X	X	X
Quality Metric Patient Satisfaction Survey												X	X	X

Outcome measures

Primary Outcome Measure

Regimen tolerability, as defined by leukopenia with an accompanying dose reduction (primary outcome), will be assessed using specific criteria. This includes a total WBC count of less than 3,000 cells/mm³ along with a reduction in the total daily dose of mycophenolate below 1500 mg per day or VGC below 900 mg daily (adjusted for renal function). This outcome will be evaluated as a dichotomous outcome (yes/no) for each patient and as a rate (number of unique leukopenia events per patient-year). Events will be considered unique once the WBC count recovers to the normal range on two consecutive measures. The length of time a patient has had leukopenia and the severity of leukopenia (nadir WBC) will also be assessed. This endpoint evaluation will occur at 3, 6, and 12 months post-transplant.

Secondary Outcome Measures

Neutropenia (secondary outcome) is defined as an absolute neutrophil count of <1,000 cells/mm³. This will be assessed as a dichotomous outcome (yes/no) per patient as well as a rate (number of unique neutropenic events per patient-year). Events will be considered unique once the neutrophil count recovers to the normal range on two consecutive measures while not receiving G-CSF therapy. The length of time a patient has neutropenia and the severity of leukopenia (nadir WBC) will also be assessed. This endpoint evaluation will occur at 3-, 6-, and 12-month post-transplant.

Mycophenolate mofetil dose reduction (secondary outcome) is defined as a reduction in dose of < 1,500 mg per day (<750 mg PO BID). This will be assessed as a dichotomous outcome (yes/no) per patient as well as a rate (number of unique mycophenolate dose reduction events per patient-year). Events will be considered unique once the mycophenolate dose is returned to the full dose for at least one week. The length of time a patient has had their mycophenolate dose < 1,500 mg per day and the severity of mycophenolate dose reduction (nadir dose) will also be assessed. This endpoint evaluation will occur at 3-, 6-, and 12-month post-transplant.

VGC dose reduction (secondary outcome) is defined as a reduction in dose of < 900 mg per day (adjusted for renal function). This will be assessed as a dichotomous outcome (yes/no) per patient as well as a rate (number of unique VGC dose reduction events per patient-year). Events will be considered unique once the VGC dose is returned to full dose for at least one week. The length of time a patient has their VGC dose reduced, and the severity of dose reduction (nadir dose) will also be assessed. This endpoint evaluation will occur at 3-, 6-, and 12-month post-transplant.

CMV prophylaxis drug dose adjustment or discontinuation (secondary outcome) is defined as a reduction in dose, holding, or discontinuation of the maribavir or VGC therapy. This will be assessed as a dichotomous outcome (yes/no) per patient as well as a rate (number of unique dose adjustment events per patient year). Events will be considered unique once the CMV prophylaxis drug dose is returned to full dose for at least one week. The length of time a patient has their CMV prophylaxis drug dose reduced or held and the severity of dose reduction (nadir dose) will also be assessed. This endpoint evaluation will occur at 3- and 6-months post-transplant.

Acute rejection (secondary outcome) is defined as a renal allograft biopsy demonstrating at least grade 1A rejection by Banff criteria. We will also record and assess all borderline acute rejections and antibody-mediated rejections using the most current Banff classification system. All patients will be required to have biopsy confirmation of rejection episodes, as per SoC and the Medical University of South Carolina (MUSC) clinical protocol. It is standard care that all kidney allograft biopsies performed for transplant recipients occur at the transplant center (study institution). Biopsies will be read by the local pathologist as usual care. The study coordinator capturing clinical event data will review the medical record to determine the timing, severity, treatment regimen, and reversibility of each acute rejection episode for all patients in the study.

Hospitalizations (secondary outcome) are defined as any admission to a hospital with at least one overnight stay. All-cause hospitalization will be assessed for this study. Hospitalizations due to CMV and other infections will be sub-analyzed as well.

Emergency department (ED) visits (secondary outcome) are defined as any visit to the ED for any cause during the post-transplant period. We will collect the cause and date of the ED visit as well. ED visits due to CMV and other infections will be sub-analyzed as well.

Efficacy Measures

CMV infection (secondary outcome) is defined as detectable CMV in the patient’s plasma. Currently, MUSC uses CMV DNA PCR (plasma IU/mL) to assess for infection with nucleic acid amplification techniques that are calibrated to the WHO international standard for human CMV. The lower level of detection is 50 IU/mL. Per the MUSC CMV protocol and SoC, any level > 1,000 IU/mL will be considered a CMV infection for the purposes of this study. All cases of CMV infection, regardless of the presence or absence of symptoms, will be classified as infection. CMV DNA PCR is measured at MUSC per our protocol using event-driven criteria, which include either leukopenia or symptoms consistent with CMV infection (N/V/D, anorexia, fever, chills, and/or malaise). For the purposes of this study, the reason for measuring CMV DNA PCR will be captured and analyzed as well. Further, the MUSC SoC CMV protocol suggests measuring CMV DNA PCR in those who are D+/R- after prophylaxis is discontinued (post-transplant months 7, 9, and 12). The protocol also recommends measuring CMV DNA PCR at months 4, 7, and 12 after discontinuation of antiviral prophylaxis in moderate-risk patients (R+). CMV symptoms and treatment will also be recorded. Late CMV infection will be defined based on the occurrence of the first CMV infection after completion of CMV prophylaxis (3- or 6-month post-transplant). These endpoints will be assessed at 3-, 6-, and 12-months post-transplant. Breakthrough cases of CMV infection, defined as the presence of CMV DNA while on anti-CMV prophylaxis, will also be assessed [22-25].

CMV disease (secondary outcome) is defined as the presence of organ dysfunction in the setting of CMV infection with biopsy-proven presence of CMV in the affected organ, done at MUSC using DNA hybridization. This will be sub-classified by organ type; the most common in kidney transplant recipients will be CMV gastrointestinal disease, CMV pneumonia, or CMV nephritis. Less common include CMV hepatitis, CMV retinitis, CMV encephalitis, CMV cystitis, CMV myocarditis, and CMV pancreatitis [22]. Late CMV disease will be defined based on the occurrence of the first CMV infection after completion of CMV prophylaxis (3- or 6-month post-transplant). This endpoint evaluation will occur at 3-, 6-, and 12-months post-transplant. Breakthrough cases of CMV disease will also be assessed.

Data collection plan

Baseline, 1-month, 2-month, 3-month, 4-month, 5-month, 6-month, 9-month, and 12-month assessment results will be collected and documented on electronic case report forms (CRFs). The data collection will be conducted by trained investigators and coordinators. They will also collect information from hospitalizations and ED visits as they occur. The principal investigator will assess adverse events, serious adverse events, and anticipated and unanticipated drug-related events as they occur.

Data management

Data capture will be accomplished using an electronic extraction and a study coordinator for manual chart abstraction. Data will be collected in a comprehensive longitudinal manner by review of the patient’s electronic and paper medical records. Data collection will include all baseline donor/recipient demographics and transplant characteristics, as well as longitudinal collection of medications, laboratory data, and clinical events during the post-transplant timeframe. The Research Electronic Data Capture (REDCap) system will be used for data management. REDCap is a secure, web-based application designed exclusively to support data capture for research studies. REDCap provides an intuitive interface for data entry (with data validation); audit trails for tracking data manipulation and export procedures; automated export procedures for seamless data downloads to common statistical packages (SPSS, SAS, Stata, and R); procedures for importing data from external sources; and advanced features, such as branching logic and calculated fields. The REDCap project was initiated at Vanderbilt University and includes more than 70 active institutional partners from Central Tibetan Schools Administration (CTSA), General Clinical Research Center (GCRC), and Research Centers in Minority Institutions (RCMI)-funded institutions, including MUSC, and others through a collaborative international consortium [26].

Data access

Any data required to support the protocol can be accessed upon request.

Oversight and Monitoring

The terms "adverse event" (AE), "serious adverse event" (SAE), "adverse drug reaction" (ADR), "fatal," "life threatening," "disability," "congenital anomaly," "hospitalization," "medically important," "association with the use of drug," and "unexpected adverse drug experience" shall have the meaning as defined in 21 CFR 312.32, as detailed below. 

Serious Adverse Event or Serious Suspected Adverse Reaction

An adverse event or suspected adverse reaction is considered "serious" if, in the view of either the investigator or sponsor, it results in any of the following outcomes: death, a life-threatening adverse event, inpatient hospitalization, prolongation of existing hospitalization, a persistent or significant incapacity, substantial disruption of the ability to conduct normal life functions, or a congenital anomaly/birth defect. Important medical events that may not result in death, be life-threatening, or require hospitalization may be considered serious when, based upon appropriate medical judgment, they may jeopardize the patient or subject and may require medical or surgical intervention to prevent one of the outcomes listed in this definition. Examples of such medical events include allergic bronchospasm requiring intensive treatment in an ED or at home, blood dyscrasias or convulsions that do not result in inpatient hospitalization, or the development of drug dependency or drug abuse.

Suspected Adverse Reaction

Any adverse event for which there is a reasonable possibility that the drug caused the adverse event. For the purposes of IND safety reporting, "reasonable possibility" means there is evidence to suggest a causal relationship between the drug and the adverse event. Suspected adverse reaction implies a lesser degree of certainty about causality than adverse reaction, which means any adverse event caused by a drug.

Unexpected Adverse Event or Unexpected Suspected Adverse Reaction

An adverse event or suspected adverse reaction is considered "unexpected" if it is not listed in the investigator brochure or is not listed at the specificity or severity that has been observed; or, if an investigator brochure is not required or available, is not consistent with the risk information described in the general investigational plan or elsewhere in the current application, as amended. For example, under this definition, hepatic necrosis would be unexpected (by virtue of greater severity) if the investigator's brochure referred only to elevated hepatic enzymes or hepatitis. Similarly, cerebral thromboembolism and cerebral vasculitis would be unexpected (by virtue of greater specificity) if the investigator's brochure listed only cerebral vascular accidents. "Unexpected," as used in this definition, also refers to adverse events or suspected adverse reactions that are mentioned in the investigator's brochure as occurring with a class of drugs or as anticipated from the pharmacological properties of the drug but are not specifically mentioned as occurring with the particular drug under investigation.

A product quality issue (PQI) is defined as any defect related to the safety, identity, strength, quality, or purity of a medicinal product or with the physical characteristics, packaging, labeling, or design of a medicinal product.

A special situation report (SSR) is defined as any of the following events: pregnancy: any case in which a pregnant patient is exposed to a medicinal product or in which a female patient or female partner of a male patient becomes pregnant following treatment with a medicinal product. Exposure is considered either through maternal exposure or via semen following paternal exposure. Breastfeeding: infant exposure from breast milk; Overdose: including any accidental or intentional overdose; drug abuse, misuse, or medication error (potential or actual); suspected (in the sense of confirmed or potential) transmission of an infectious agent by a medicinal product; lack of efficacy of a medicinal product; accidental exposure; use outside the terms of the marketing authorization, also known as “off-label” use; use of a falsified medicinal product; unintended benefit. An SSR should be reported even if there is no associated AE.

Reporting responsibilities

As the sponsor of the study, MUSC and/or the PI shall be solely responsible for reporting any and all serious and unexpected adverse drug experiences or other safety information associated with the use of the study drug to the applicable regulatory authorities, the institutional review board (IRB) or independent ethics committee (IECs), and any other investigators, as required by applicable laws and regulations, including 21CFR312.32, within the timelines required, and whether or not the study is being performed under an IND.

MUSC and/or the PI must notify Takeda (Tokyo, Japan) or its designee within one (1) working day of becoming aware of a fatal or life-threatening SAE, within four (4) calendar days for other SAEs, and within seven (7) calendar days for all other events/issues listed above. This is achieved by submitting an AE Report Form to TAKEDA’s Pharmacovigilance Department at PVSafetyAmericas@takeda․com. 

MUSC and/or the PI may be contacted by Takeda or its designee to obtain additional information on an adverse event or for data clarification. MUSC and/or the PI shall use its best efforts to obtain the requested additional information and will notify Takeda or its designee within one (1) working day of obtaining the additional information for a fatal or life-threatening SAE, within four (4) calendar days for other SAEs, and within seven (7) calendar days for all other events/issues listed above. 

MUSC and/or the PI agree to update the protocol and the informed consent at the request of Takeda for safety-related reasons.

Data analysis

The analysis to assess objectives for the study will be conducted using standard and widely accepted statistical methods. Data will be reported using percentages for nominal variables and univariate comparisons using Fisher’s exact test or Pearson’s chi-squared test as appropriate. This includes baseline demographics and transplant characteristics, as well as the non-time-to-event outcome variables. For continuous variables with normal distribution, results will be reported using means and standard deviations with univariate statistical comparison using Student’s t-test for two independent samples. For non-normally distributed variables, data will be reported using medians and interquartile ranges, with univariate statistical comparison conducted using the Mann-Whitney U test. Normal distribution of continuous variables will be assessed using normality plots and the Shapiro-Wilk test. Normal variance will be assessed using Levene’s test for equality of variances. Chi-square or Fisher’s exact test will be used for univariate assessments of key outcomes, including the incidence of leukopenia, neutropenia, CMV antiviral prophylaxis dosing interruption, CMV infection, acute rejection, graft loss, and death.

If necessary, multivariable analysis assessments for objectives 1 and 2 will be conducted using Cox regression time-to-event models. The primary variable of interest in these models will be treatment (maribavir vs. valganciclovir). If necessary and appropriate, given event rates, Cox regression analysis will also be used for survival analyses involving all time-to-event outcomes, including time to acute rejection, time to graft loss, and time to death. Models will be adjusted for key variables known to influence outcomes, including CMV serostatus and rATG induction. To prevent overfitting, no more than one covariate for every five events will be included in these models. Given the small sample size and expected event rates, Cox models will likely be overfit if including more than 2 covariates plus the primary treatment variable. Thus, multivariable modeling will be limited to only include key baseline variables that are known to influence the risk of CMV infection and that significantly differ by treatment arm based on preliminary univariate analyses; this will most likely only include CMV D+/R- serostatus and/or rATG induction. Prior to conducting Cox regression, modeling assumptions will be assessed, including proportionality of hazards across treatment arms and assessing the optimal functional forms of non-dichotomous covariates.

For aim 3, total hospital charges and healthcare utilization will be modeled using a validated method we have used in previous studies. This will be a two-part statistical model for zero-heavy continuous data. The general format of the suggested model in which we fit a logistic model for the probability of a non-zero response and a conditional generalized linear model (GLM) for the mean response given that it is non-zero. We will consider several distributions for the GLM part (e.g., log-normal, gamma, Weibull, and zero-inflated negative binomial [ZINB]), and the best-fitting model will be selected using the Bayesian information criterion (BIC). Parameter estimates of percent change in total hospital charge per unit increase in the values of each covariate in the model and their 95% CI will be computed using SAS Proc FMM [25].

For aim 4, survey results will be aggregated using validated methods for each survey. The mean scores will be compared between treatment arms, as well as the mean change across the three measurement timepoints. Comparisons will be made using standard statistical tests based on the distribution of the results (Student’s t-test for normal variance or the Mann-Whitney test for non-normally distributed data). Mean change will be assessed using general linear modeling (GLM) for repeated measures data with the appropriate link based on the distribution of the measures. 

For aim 5, interaction terms will be added to multivariable models (Cox regression or GLM) to assess if there are differences by race (race treatment) or sex (sex treatment). A p-value of <0.1 will be considered sufficient evidence of likely effect modification, and the analysis will then be stratified by the appropriate variable (either race or sex) for a given outcome if there is in fact a significant interaction. Given the limited sample size, there is likely low power to detect statistically significant differences for this aim; thus, aim 5 is considered exploratory in nature.

We will also assess for any impact of maribavir on tacrolimus dosing or trough concentrations. Steady-state stable doses of tacrolimus (mg/day) and whole blood trough concentrations will be assessed and compared in patients during maribavir therapy versus after completion of therapy. Changes will be compared between the treatment arms using the difference-in-difference methodology to assess drug-drug interactions. The level-normalized dose (dose needed to achieve a level of 8 ng/mL using linear proportions) will also be compared using difference-in-difference analysis. Finally, the proportion of patients in the maribavir arm that have more than a 10% change in level-normalized dose after discontinuing maribavir therapy will be collected and reported. These analyses are considered exploratory.

## Results

The trial is in the follow-up and data analysis phase. The first patient was recruited in November 2023, and enrollment was completed at the end of June 2024.

## Discussion

The main goal of this study is to assess the tolerability of maribavir versus valganciclovir (VGC) prophylaxis in adult kidney transplant recipients at high risk of CMV infection, which we defined as the rates of clinically significant leukopenia, as this is the most common and problematic issue that arises in kidney transplant recipients requiring CMV antiviral prophylaxis. Previous research has shown that these medications are equally effective at preventing and treating CMV infection, but there is limited data that assesses the tolerability of these medications in adult kidney transplant recipients. This study is novel in that it is specifically designed to assess regimen tolerability as the primary outcome, and efficacy measures are secondary outcomes. This was intentional, as there is sufficient data to suggest regimen efficacy will be similar between antiviral therapies, and focusing on patient-reported outcomes and tolerability seemed more relevant than a non-inferiority study. Given the potential differences in costs, another key measure will be to assess healthcare utilization, as increased rates of leukopenia can lead to increased use of labs, clinic visits, and provider time. 

## Conclusions

The trial is in the follow-up and data analysis phase. The first patient was recruited in November 2023, and enrollment was completed at the end of June 2024.
